# Moderation of thyroid hormones for the relationship between amyloid and tau pathology

**DOI:** 10.1186/s13195-024-01534-4

**Published:** 2024-07-23

**Authors:** Jeong Hyeon Byeon, Min Soo Byun, Dahyun Yi, Joon Hyung Jung, Bo Kyung Sohn, Yoon Young Chang, Nayeong Kong, Gijung Jung, Hyejin Ahn, Jun-Young Lee, Yun-Sang Lee, Yu Kyeong Kim, Dong Young Lee, for the KBASE Research Group, Chul-Ho Sohn, Inhee Mook- Jung, Murim Choi, Yu Jin Lee, Seokyung Hahn, Hyun Jung Kim, Mun Young Chang, Seung Hoon Lee, Na Young Han, Jisoo Pae, Hansoo Park, Jee Wook Kim, Jong-Min Lee, Dong Woo Lee, Seok Woo Moon, Hyewon Baek, Yoon-Keun Kim, Jong-Won Kim, Seung-Ho Ryu, Shin Gyeom Kim, Jong Inn Woo, Sang Eun Kim, Gi Jeong Cheon, Koung Mi Kang, Jee-Eun Park, Hyeong Gon Yu, Hyo Jung Choi, Young Min Choe, Kwangsoo Kim, So Yeon Jeon, Woo Jin Kim, Kang Ko, Jun Ho Lee, Sung Wook Park, Haejung Joung, Han Na Lee, Gihwan Byeon, Kiyoung Sung, Dong Kyun Han, Seung Min Han, Min Jung Kim, Min Jae Kim, Seo Hee Park, Mimi Kim, Woojin Cha, Hyeryeon Yeom, Musung Keum, Min Jeong Kim, Donghee Kim, Kyungtae Kim, Jeongmin Choi, Hye Ji Choi, Bae Han Sol, Dohyun Woo, Seunghyuk Ha

**Affiliations:** 1 Department of Neuropsychiatry, Seoul National University Hospital, 101 Daehak-Ro, Jongno-Gu, Seoul, 03080 Republic of Korea; 2 Department of Psychiatry, Seoul National University College of Medicine, 101 Daehak-Ro, Jongno-Gu, Seoul, 03080 Republic of Korea; 3 Institute of Human Behavioral Medicine, Medical Research Center, Seoul National University, Seoul, Republic of Korea; 4 Department of Psychiatry, Chungbuk National University Hospital, Cheongju, Republic of Korea; 5 Department of Psychiatry, Inje University Sanggye Paik Hospital, Seoul, Republic of Korea; 6 Department of Psychiatry, Keimyung University Hospital, Daegu, Republic of Korea; 7 Interdisciplinary Program of Cognitive Science, Seoul National University, Seoul, Republic of Korea; 8 Department of Neuropsychiatry, SMG-SNU Boramae Medical Center, Seoul, Republic of Korea; 9 Department of Nuclear Medicine, Seoul National University College of Medicine, Seoul, Republic of Korea; 10 Department of Nuclear Medicine, SMG-SNU Boramae Medical Center, Seoul, Republic of Korea

**Keywords:** Thyroid hormone, Thyroid-stimulating hormone, Alzheimer’s disease, Amyloid beta, Tau, Positron emission tomography

## Abstract

**Background:**

Altered thyroid hormone levels have been associated with increased risk of Alzheimer's disease (AD) dementia and related cognitive decline. However, the neuropathological substrates underlying the link between thyroid hormones and AD dementia are not yet fully understood. We first investigated the association between serum thyroid hormone levels and in vivo AD pathologies including both beta-amyloid (Aβ) and tau deposition measured by positron emission tomography (PET). Given the well-known relationship between Aβ and tau pathology in AD, we additionally examined the moderating effects of thyroid hormone levels on the association between Aβ and tau deposition.

**Methods:**

This cross-sectional study was conducted as part of the Korean Brain Aging Study for Early Diagnosis and Prediction of Alzheimer's Disease (KBASE) cohort. This study included a total of 291 cognitively normal adults aged 55 to 90. All participants received comprehensive clinical assessments, measurements for serum total triiodothyronine (T3), free triiodothyronine (fT3), free thyroxine (fT4), and thyroid-stimulating hormone (TSH), and brain imaging evaluations including [^11^C]-Pittsburgh compound B (PiB)- PET and [^18^F] AV-1451 PET.

**Results:**

No associations were found between either thyroid hormones or TSH and Aβ and tau deposition on PET. However, fT4 (*p* = 0.002) and fT3 (*p* = 0.001) exhibited significant interactions with Aβ on tau deposition: The sensitivity analyses conducted after the removal of an outlier showed that the interaction effect between fT4 and Aβ deposition was not significant, whereas the interaction between fT3 and Aβ deposition remained significant. However, further subgroup analyses demonstrated a more pronounced positive relationship between Aβ and tau in both the higher fT4 and fT3 groups compared to the lower group, irrespective of outlier removal. Meanwhile, neither T3 nor TSH had any interaction with Aβ on tau deposition.

**Conclusion:**

Our findings suggest that serum thyroid hormones may moderate the relationship between cerebral Aβ and tau pathology. Higher levels of serum thyroid hormones could potentially accelerate the Aβ-dependent tau deposition in the brain. Further replication studies in independent samples are needed to verify the current results.

**Supplementary Information:**

The online version contains supplementary material available at 10.1186/s13195-024-01534-4.

## Background

The relationship between thyroid hormones and cognitive function is well-recognized [1–3]. Furthermore, thyroid dysfunctions or alterations in thyroid hormone levels have been associated with an increased risk of Alzheimer's disease (AD) dementia and related cognitive decline [4–8]. For instance, a study based on the Rotterdam Study revealed that the risk of AD dementia increases by 4% for every 1 pmol/L rise in free thyroxine (fT4) [5]. Additionally, another clinical investigation demonstrated that individuals with thyroid-stimulating hormone (TSH) levels below 0.4 μIU/ml face a 3.5-fold higher risk of AD dementia [8].

However, the neuropathological substrates that underlie the link between thyroid hormones and AD dementia are not yet fully understood. In terms of beta-amyloid (Aβ) deposition, a key pathology of AD, several clinical studies have reported that lower fT4 levels were associated with an increase of in vivo cerebral Aβ deposition as observed on positron emission tomography (PET) [9, 10]. In contrast to this, however, other clinical studies reported no association between serum thyroid hormones and cerebrospinal fluid (CSF) Aβ_1−42_ [11] or Aβ deposition on PET [12], and a post-mortem study even demonstrated that individuals with higher total T4 levels had increased amyloid plaques in the brain [13].

Moreover, limited Information is currently available about the relationship between thyroid hormones and brain tau deposition, another key pathology of AD: While a cross-sectional study with a small population (comprising 36 euthyroid AD dementia patients and 34 healthy controls) reported no association between serum thyroid hormones and CSF tau levels [11], no other studies have yet investigated the association of serum thyroid hormones with in vivo brain tau deposition as measured by PET examination. Given that brain tau deposition is more closely correlated with cognitive decline than Aβ deposition [14, 15], information about the relationship between thyroid hormones and tau pathology is invaluable for understanding the pathological substrates linking thyroid function with AD-related cognitive decline and for suggesting potential targets for therapeutic intervention against AD within the AD research community.

Therefore, we initially investigated the association between serum thyroid hormone levels and in vivo AD pathologies, including both Aβ and tau deposition measured by PET, in cognitively healthy older adults. Moreover, given the well-known relationship between Aβ and tau in AD [16], we examined the moderating effects of thyroid hormone levels on the association between Aβ and tau deposition. Although no previous studies have reported the moderation effects of thyroid hormones on the relationship between Aβ and tau, a PET study involving non-demented older adults suggested an interaction between thyroid hormones and Aβ deposition on subsequent neurodegeneration [12].

## Methods

### Participants

This study was conducted as part of the Korean Brain Aging Study for Early Diagnosis and Prediction of Alzheimer’s Disease (KBASE) cohort. KBASE is a cohort study with the aim of discovering new biomarkers for Alzheimer's disease (AD) and exploring lifetime experiences or physical changes that may impact the brain pathology associated with the AD process [17]. Participants were recruited from two university hospitals in Seoul, South Korea, namely Seoul National University Hospital (SNUH) and Seoul National University-Seoul Metropolitan Government (SNU-SMG) Boramae Medical Center, as well as two public dementia centers. The present study included 291 cognitively normal (CN) individuals aged 55 to 90. All participants had a Clinical Dementia Rating (CDR) [18] score of 0, and were not diagnosed with mild cognitive impairment (MCI) [19] or dementia [20]. The exclusion criteria included 1) major psychiatric illnesses such as schizophrenia, bipolar disorder, major depressive disorder, alcohol/substance abuse or dependence, delirium; 2) significant neurological or medical conditions that could impact mental function; 3) contraindications for MRI; 4) illiteracy; 5) significant visual/hearing impairment, communication issues, or behavioral problems hindering the assessment; and 6) those currently taking an investigational drug. The research protocol was approved by the Institutional Review Boards of SNUH and SNU-SMG Boramae Medical Center, and written informed consent was obtained from all participants.

### Clinical assessment

All participants were evaluated by trained psychiatrists according to the KBASE clinical assessment protocol [17], which includes the Korean version of the Consortium to Establish a Registry for Alzheimer’s Disease assessment packet (CERAD-K) [21]. The evaluation process involved reliable informants and a thorough review of medical records to ensure reliability. Depressive symptoms were assessed by the Korean version of the Geriatric Depression Scale (GDS-KR) [22]. The Vascular Risk Score (VRS), which ranges from 0 to 6, was calculated by summing the number of vascular risk factors, including hypertension, diabetes mellitus, hyperlipidemia, coronary heart disease, stroke, and transient ischemic attack [23]. The CERAD-K neuropsychological tests were also administered by trained neuropsychologists [24].

### Laboratory assessment

Blood samples were collected after an overnight fast. Total triiodothyronine (T3), free triiodothyronine (fT3), free thyroxine (fT4), and thyroid stimulating hormone (TSH) were measured using the chemiluminescence immunoassay on the ADVIA Centaur XP system (Siemens, Washington DC, USA). The normal ranges for each hormone were established as follows: T3 is 65–150 ng/dl, fT3 is 2.3–4.2 pg/ml, fT4 is 0.89–1.76 ng/dl, and TSH is 0.55–4.78 μIU/ml. In addition, apolipoprotein E (APOE) genotyping was performed on blood samples [25], with individuals possessing one or more APOE ε4 (APOE4) alleles being classified as APOE4 positive.

### Measurement of cerebral Aβ and tau deposition

At the time of clinical evaluation, participants underwent simultaneous three-dimensional [^11^C]-Pittsburgh compound B (PiB)-PET and 3D T1-weighted MRI scans using a 3.0 T Biograph mMR (PET-MR) scanner (Siemens, Washington DC, USA) to measure cerebral Aβ deposition. Detailed explanations of the process for obtaining and processing PiB-PET images can be found in our previous report [26]. The autonomic anatomical labeling algorithm and region combining method were used to set the regions of interest (ROIs) [27, 28]. The frontal, lateral parietal, posterior cingulate-precuneus, and lateral temporal cortex were chosen as ROIs, given previous reports of prominent Aβ deposition in the four areas [29]. For the quantitative normalization of cerebral PiB uptake values, the cerebellar gray matter, known for its relatively low Aβ deposition, was set as the reference region [30]. The global cortical standardized uptake value ratio (SUVR) was calculated by dividing the mean value of all voxels in the mentioned 4 ROIs by the mean cerebellar uptake value, and it was regarded as indicative of global cerebral Aβ deposition. We used a global cortical SUVR cutoff of 1.21 to determine amyloid positivity [31].

Additionally, a subset of participants (n = 74) underwent [^18^F] AV-1451 PET scans using a Biograph True point 40 PET/CT scanner (Siemens, USA) to measure cerebral tau deposition, on average 2.45 years (SD 0.35) after clinical evaluation and PiB-PET imaging. Detailed descriptions of the specific methods and processing procedures for obtaining AV-1451 PET images are provided in our previous report [26]. AV-1451 PET images were normalized by the mean inferior cerebellar gray matter uptake, according to the published code [32]. The partial volume corrected AV-1451 SUVR of the inferior temporal (IT) ROI was quantified to estimate cerebral tau deposition. IT region is the neocortical site of tau deposition in early stage of AD [33, 34]. Tau positivity was defined as follows: participants classified as Alzheimer's disease Braak stages 0-II were considered negative, while those in Braak stages III-VI were considered positive [34, 35].

### Statistical analysis

Partial correlation analyses were conducted to investigate the association between thyroid hormones (or TSH) and global Aβ (or tau) deposition, with age, sex, and APOE4 positivity as covariates. We performed multiple linear regression analyses to examine the moderating effects of thyroid hormones and TSH on the association between Aβ and tau deposition. These analyses included thyroid hormones (or TSH) x global Aβ deposition interaction term as well as thyroid hormones (or TSH) and global Aβ deposition as independent variables, with IT tau deposition as a dependent variable and age, sex, and APOE4 positivity as covariates. If the interaction effects were statistically significant, we conducted subsequent subgroup analyses. Subgroups were created based on median values of each hormone. We than conducted multiple linear regression analyses, adjusting for age, sex, and APOE4 positivity, to assess the effect of global Aβ deposition on IT tau deposition. In all statistical analyses, two-tailed *p*-values < 0.05 were considered as statistically significant. IBM SPSS Statistics 26 (IBM Corporation, Armonk, NY, USA) was used to conduct the analyses.

## Results

### Demographic and clinical characteristics

The demographic and clinical characteristics of the study participants are summarized in Table 1. A total of 291 participants (mean [SD] age, 69.11 [8.06] years; 151 women [51.9%]) were included in the study. Out of the total participants, 6 (2%) had subclinical hyperthyroidism, 19 (7%) had subclinical hypothyroidism, and 6 (2%) had TSH within the normal range but low fT4, indicating secondary hypothyroidism, nonthyroidal illness, or physiological aging [36, 37].

**Table 1:** Demographic and clinical characteristics of the tau PET subset and all participants

Characteristics	All participants (n = 291)	Participants with tau PET (n = 74)
Age (years)	69.11 (8.06)	69.62 (7.47)
Education (years)	11.84 (4.83)	11.50 (4.34)
Gender, female (%)	151 (51.9)	47 (63.5)
CDR	0	0
APOE ε4 allele( +) (%)	54 (18.6)	14 (18.9)
GDS	4.74 (4.96)	5.18 (4.96)
VRS	1.05 (0.96)	1.11 (0.99)
Neuropsychological tests		
MMSE-KC score	26.95 (2.46)	27.16 (2.11)
CERAD-K total score	72.99 (10.33)	72.61 (9.87)
Global Aβ deposition (SUVR)	1.20 (0.22)	1.31 (0.35)
Amyloid positivity (%)	53 (18.2)	23 (31.1)
IT tau deposition (SUVR)	1.43 (0.27)	1.43 (0.27)
Tau positivity (%)	18 (6.2)	18 (24.3)
T3 (mg/dl)	103.91 (17.90)	103.31 (17.60)
TSH (μIU/ml)	2.37 (2.14)	2.22 (1.54)
fT4 (ng/dl)	1.20 (0.18)	1.14 (0.16)
fT3 (pg/ml)	3.13 (0.36)	3.07 (0.34)

*Notes*: Data for continuous variables are presented as a mean (SD). Categorical variables are presented as N (%)

*Abbreviations*: *PET* positron emission tomography, *CDR* Clinical Dementia Rating, *APOE* Apolipoprotein E, *GDS* Geriatric Depression Scale, *VRS* Vascular Risk Score, *MMSE-KC* Mini-Mental State Examination in the Korean version of CERAD Assessment Packet, *CERAD-K* Korean version of the Consortium to Establish a Registry for Alzheimer’s Disease Assessment Packet, *Aβ* beta-amyloid, *SUVR* standardized uptake value ratio, *IT* inferior temporal, *T3* triiodothyronine, *T4* thyroxine, *TSH* thyroid-stimulating hormone

### Association between serum thyroid hormones and Aβ and tau

There were no significant associations of global Aβ and IT tau deposition with the serum levels of thyroid hormones and TSH (Table 2, Figure S1).

**Table 2:** Partial correlation between serum thyroid hormones and Aβ deposition, and tau deposition

		T3	TSH	fT4	fT3
Aβ deposition	correlation	0.077	-0.009	-0.058	-0.005
(n = 291)	*p*-value	0.192	0.881	0.325	0.939
Tau deposition	correlation	0.090	-0.213	-0.013	-0.067
(n = 74)	*p*-value	0.455	0.075	0.917	0.577

*Notes*: Partial correlation analysis used age, sex, and APOE ε4 positivity as the covariates

*Abbreviations*: *Aβ* beta-amyloid, *T3* triiodothyronine, *T4* thyroxine, *TSH* thyroid-stimulating hormone, *APOE* Apolipoprotein E

### Moderation effects of thyroid hormones on the relationship between Aβ and tau

**Fig. 1:**
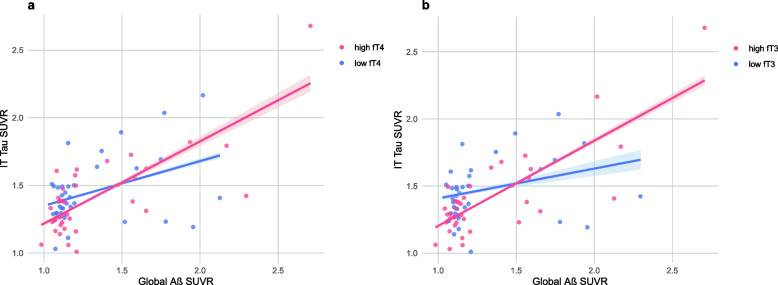
Moderating effects of thyroid hormones on the relationships between Aβ and tau deposition. Notes: To effectively demonstrate the moderating effects, participants were divided into two subgroups based on thyroid hormone levels. Each line represents a regression line for IT tau deposition, with global Aβ deposition as the independent variable, controlling for age, sex, and APOE ε4 positivity. The shaded regions demonstrate the 95% confidence intervals of the regression lines. Abbreviations: Aβ beta-amyloid, IT inferior temporal, SUVR standardized uptake value ratio, T3 triiodothyronine, T4 thyroxine, APOE Apolipoprotein E

There was a significant serum fT4 x global Aβ deposition interaction on IT tau (*p* = 0.002) (Table 3). Further separate analyses on the two subgroups, divided by the median value of fT4, demonstrated a stronger association between Aβ and tau in the higher fT4 group compared to the lower group (Table 4, Fig. 1a). Similarly, a significant interaction effect was observed between serum fT3 and Aβ deposition on IT tau deposition (*p* = 0.001) (Table 3). Subsequent subgroup analyses showed a stronger relationship between Aβ and tau in the higher fT3 group compared to the lower group (Table 4, Fig. 1b). There were no significant differences in characteristics between fT4 or fT3 subgroups, as summarized in Table S1 and Table S2. Neither T3 nor TSH showed significant interaction effect with Aβ on tau deposition (Table 3).

**Table 3:** Interaction effects of thyroid hormones with Aβ deposition on tau deposition (n = 74)

	B (95% CI)	SE	*p*-value
Model 1^a^			
Global Aβ deposition	0.477 (0.314–0.640)	0.082	0.000
Model 2^b^			
Global Aβ deposition x T3	0.002 (-0.008–0.011)	0.005	0.707
Global Aβ deposition x TSH	-0.072 (-0.153–0.008)	0.040	0.078
Global Aβ deposition x fT4	1.022 (0.375–1.668)	0.324	0.002
Global Aβ deposition x fT3	0.714 (0.307–1.121)	0.204	0.001

*Notes*: ^a^ A multiple linear regression model for IT tau deposition included Global Aβ deposition as an independent variable, after adjusting for age, sex, and APOE ε4 positivity

^b^ Multiple linear regression models for IT tau deposition included serum thyroid hormones and global Aβ as interactive predictors, after adjusting for age, sex, and APOE ε4 positivity

Summary of the model: IT tau ~ Global Aβ deposition x Thyroid hormone + Thyroid hormone + Global Aβ deposition + age + sex + APOE ε4 positivity

*Abbreviations*: *Aβ* beta-amyloid, *T3* triiodothyronine, *T4* thyroxine, *TSH* thyroid-stimulating hormone, *IT* inferior temporal, *APOE* Apolipoprotein E, *CI* confidence interval, *SE* standard error

**Table 4:** Relationship between Aβ and tau deposition in subgroups based on thyroid hormone levels (n = 74)

	B (95% CI)	SE	R^2^	*p*-value
Low fT4 (fT4 < 1.12)	0.326 (-0.001–0.653)	0.161	0.182	0.051
High fT4 (fT4 ≥ 1.12)	0.547 (0.353–0.742)	0.096	0.611	0.000
Low fT3 (fT3 < 3.05)	0.173 (-0.076–0.421)	0.122	0.215	0.167
High fT3 (fT3 ≥ 3.05)	0.694 (0.499–0.889)	0.096	0.659	0.000

*Notes*: Multiple linear regression models for IT tau deposition included global Aβ deposition as an independent variable, after adjusting for age, sex, and APOE ε4 positivity

*Abbreviations*: *Aβ* beta-amyloid, *T3* triiodothyronine, *T4* thyroxine, *IT* inferior temporal, *APOE* Apolipoprotein E, *CI* confidence interval, *SE* standard error

### Sensitivity analyses results

Sensitivity analyses conducted solely for participants in a euthyroid state revealed results (Tables S3 and S4) similar to those from the main analyses. Additionally, sensitivity analyses including VRS as an additional covariate did not change the results (Tables S5 and S6).

When the outlier in the upper right corner of Fig. 1 was excluded for additional sensitivity analyses, it was found that serum fT3 still significantly interacted with Aβ deposition, influencing IT tau deposition, similar to the main analyses. However, no significant interaction effect was observed between serum fT4 and Aβ deposition (Table S7). Despite the absence of a significant interaction between serum fT4 and Aβ deposition after outlier exclusion, additional subgroup analyses for low and high fT4 groups produced results that were in line with those seen in the overall sample (Table S8, Figure S2).

## Discussion

In the current study, associations between serum thyroid hormone levels and in vivo AD pathologies were investigated in clinically asymptomatic older adults. While the results showed no direct associations of thyroid hormones or TSH with cerebral Aβ and tau deposition, we found that fT4 and fT3 strengthened the positive relationship between Aβ and tau deposition.

Our finding of no association between thyroid hormones and cerebral Aβ deposition is in line with some previous reports. In one study, there was no correlation between serum thyroid hormone levels and global Aβ deposition on PET in non-demented older adults [12]. Similarly, another study found no correlation between blood thyroid hormones and CSF Aβ_1−42_ in AD patients and CN controls [11]. However, a couple of other studies showed that lower fT4 was associated with increased cerebral Aβ deposition [9, 10]. It is not easy to clearly explain the conflicting findings, they may be attributed to differences in participant characteristics including sample size, cognitive status, and comorbid medical conditions may contribute to the discrepancies. Our study had a relatively larger sample size (n = 291) than other studies, which ranged from 69 to 148 participants [9–12]. In addition, we included only CN individuals, while some other studies encompassed both cognitively impaired individuals as well as CN ones. In individuals with cognitive impairment, such as MCI and AD dementia, the neurodegeneration caused by AD process could potentially decrease the secretion of thyrotropin releasing hormone (TRH) and TSH, subsequently reducing the levels of thyroid hormones [7, 38]. This may explain in part the association between lower thyroid hormone and increased brain Aβ [9, 10].

There was no direct association between thyroid hormones and cerebral tau deposition. This aligns with a previous study which reported no association between thyroid hormones and CSF total tau and phosphorylated tau [11, 39]. However, we found significant interaction effects of fT4 and fT3 with Aβ deposition on tau deposition, which indicated that both serum fT4 and fT3 moderate the relationship between Aβ and tau deposition. The positive correlation between Aβ and tau deposition was stronger in individuals with higher serum fT4 or fT3 levels (Table 3, Table 4, and Fig. 1). In a recent study involving euthyroid non-demented adults, thyroid hormone levels were not associated with global Aβ deposition. However, higher serum levels of fT4 and fT3 were associated with decreased cortical glucose metabolism measured by [^18^F]-fluorodeoxyglucose (FDG)-PET in Aβ positive participants, but not in Aβ negative ones, indicating interaction between thyroid hormones and Aβ deposition on subsequent neurodegeneration [12]. Although no previous study has yet demonstrated moderation effects of thyroid hormones on the relationship between Aβ and tau deposition, reports for a possible interaction between thyroid hormones and Aβ or AD diagnosis on neurodegeneration may be in line with our findings for an interaction between thyroid hormones and Aβ on tau deposition, which is closely related to further neurodegeneration [40].

In sensitivity analyses, when one outlier was removed, we discovered that the interaction effect between fT4 and Aβ deposition on tau deposition was not significant, while the interaction between fT3 and Aβ deposition was significant. However, subgroup analyses based on serum fT4 levels after outlier removal showed that the correlation between Aβ deposition and tau deposition was more pronounced in the higher fT4 group compared to the lower fT4 group. Consequently, we deemed this outlier a meaningful data point and included it in the main analysis, acknowledging that its arbitrary removal could result in biased inferences about the population [41, 42]. Given that this outlier can influence the results depending on its inclusion, we reported the analysis results both with and without the outlier [41]. The possibility that the interaction effect between fT4 and Aβ deposition was a false positive cannot be dismissed, thus underscoring the need for replication studies in independent samples in the future.

The underlying mechanisms for the interaction between thyroid hormones and Aβ on tau deposition are not easily understood. Nevertheless, a few possible explanations can be provided. Firstly, the role of glutamate in Aβ toxicity is well-recognized, with reports indicating that glutamate activates tau kinase through N-methyl-D-aspartate receptors, contributing to tau phosphorylation and accumulation [43]. Although not yet conclusive, some animal studies demonstrated reduced glutamate levels in hypothyroidism, and administration of T4 increased glutamate levels [44, 45]. Other animal studies also reported that the inhibition of Na^+^/K^+^-ATPase by increased thyroid hormone led to changes in transmembrane ion gradients, resulting in decreased glutamate uptake and an increase in extracellular excitatory glutamate levels [45–47]. Taken together, thyroid hormones may facilitate the influence of Aβ on tau deposition through elevating glutamate levels. Secondly, evidence suggests that Aβ serves as a source of reactive oxygen species (ROS) and induces oxidative stress. It is also well-known that increased ROS or oxidative stress facilitate tau hyperphosphorylation [48, 49]. Given that an increased formation of ROS and oxidative stress has been observed in patients with hyperthyroidism [50, 51], elevated thyroid hormone levels may influence the process of Aβ-induced oxidative stress, leading to tau hyperphosphorylation and accumulation.

Our study found no significant association between TSH and AD pathology (Table 2, Table 3). This result aligns with previous reports that found no association between TSH and hippocampal atrophy on MRI [52], as well as no association between TSH and cerebral glucose hypometabolism in AD-signature regions [9]. As age advances, the biological activity of TSH changes and becomes independent of thyroid function [53]. Therefore, TSH may not always be an accurate reflection of thyroid function, which could contribute to the observed lack of association.

The present study has several limitations. Firstly, as this is a cross-sectional study, it is difficult to infer a causal relationship. Further longitudinal studies to investigate the association between thyroid hormones and AD pathology are needed. Secondly, this study did not include a replication cohort to validate the findings. Further replication studies are still needed to confirm the current results. Thirdly, thyroid hormone levels were measured only once in this study. Although measurements were taken at a consistent time between 9 and 10 am after fasting to reduce diurnal variation, seasonal variation of the hormones may influence the results. Fourthly, most participants in this study were in a euthyroid status, which resulted in a narrower range of thyroid hormone levels. This could potentially reduce the likelihood of detecting the association between thyroid hormone levels and AD pathology. Finally, the relatively small sample size (n = 74) for tau PET analyses might have contributed to the null finding for the direct association between thyroid hormones and tau deposition, although we found a moderation effect of thyroid hormones on the Aβ-tau relationship. Further studies with larger sample sizes are needed.

## Conclusion

The present findings suggest that serum thyroid hormones may moderate the relationship between cerebral Aβ and tau pathology. Higher levels of serum thyroid hormones, even within the normal range, could potentially accelerate Aβ-dependent tau deposition in the brain. Further replication studies in independent samples are needed to verify the current results.

### Supplementary Information

Below is the link to the electronic supplementary material.

Supplementary Material 1: Table S1. Demographic and clinical characteristics of participants according to fT4 subgroups. Table S2. Demographic and clinical characteristics of participants according to fT3 subgroups. Table S3. Sensitivity analysis: Partial correlation between serum thyroid hormones and Aβ deposition, and tau deposition in euthyroid participants. Table S4. Sensitivity analysis: Interaction effects of serum thyroid hormones with global Aβ deposition on inferior temporal tau deposition in euthyroid participants (n = 65). Table S5. Sensitivity analysis: Partial correlation between serum thyroid hormones and Aβ deposition, and tau deposition after including VRS as an additional covariate. Table S6. Sensitivity analysis: Interaction effects of serum thyroid hormones with global Aβ deposition on inferior temporal tau deposition after including VRS as an additional covariate (n = 74). Table S7. Sensitivity analysis: Interaction effects of thyroid hormones with Aβ deposition on tau deposition, excluding an outlier (n = 73). Table S8. Sensitivity analysis: Relationship between Aβ and tau deposition in subgroups based on thyroid hormone levels, excluding an outlier (n = 73). Figure S1. Scatter plots showing the partial correlation between serum thyroid hormones and Aβ deposition, and tau deposition. Figure S2. Sensitivity analysis: Moderating effects of thyroid hormones on the relationships between Aβ and tau deposition, excluding an outlier (n = 73).

## Data Availability

No datasets were generated or analysed during the current study.

## Abbreviations

AD: Alzheimer's disease
KBASE: Korean Brain Aging Study for Early Diagnosis and Prediction of Alzheimer's Disease
T3: Triiodothyronine
T4: Thyroxine
TSH: Thyroid-stimulating hormone
PiB: Pittsburgh compound B
PET: Positron emission tomography
Aβ: Beta-amyloid
CSF: Cerebrospinal fluid
SNUH: Seoul National University Hospital
SNU-SMG: Seoul National University-Seoul Metropolitan Government
CN: Cognitively normal
CDR: Clinical Dementia Rating
MCI: Mild cognitive impairment
CERAD-K: Consortium to Establish a Registry for Alzheimer’s Disease assessment packet
GDS-KR: Korean version of the Geriatric Depression Scale
VRS: Vascular Risk Score
APOE: Apolipoprotein E
APOE4: Apolipoprotein E ε4
ROI: Regions of interest
SUVR: Standardized uptake value ratio
IT: Inferior temporal
FDG: Fluorodeoxyglucose
ROS: Reactive oxygen species
MMSE-KC: Mini-Mental State Examination in the Korean version of CERAD Assessment Packet
