# Supplementary material for: Moderation of thyroid hormones for the relationship between amyloid and tau pathology
Source: Alzheimers Res Ther. 2024 Jul 23;16:164. doi: 10.1186/s13195-024-01534-4 (PMC11264392; doi:10.1186/s13195-024-01534-4)
Supplement: Supplementary file 1 — Supplementary Material 1: Table S1. Demographic and clinical characteristics of participants according to fT4 subgroups. Table S2. Demographic and clinical characteristics of participants according to fT3 subgroups. Table S3. Sensitivity analysis: Partial correlation between serum thyroid hormones and Aβ deposition, and tau deposition in euthyroid participants. Table S4. Sensitivity analysis: Interaction effects of serum thyroid hormones with global Aβ deposition on inferior temporal tau deposition in euthyroid participants (n = 65). Table S5. Sensitivity analysis: Partial correlation between serum thyroid hormones and Aβ deposition, and tau deposition after including VRS as an additional covariate. Table S6. Sensitivity analysis: Interaction effects of serum thyroid hormones with global Aβ deposition on inferior temporal tau deposition after including VRS as an additional covariate (n = 74). Table S7. Sensitivity analysis: Interaction effects of thyroid hormones with Aβ deposition on tau deposition, excluding an outlier (n = 73). Table S8. Sensitivity analysis: Relationship between Aβ and tau deposition in subgroups based on thyroid hormone levels, excluding an outlier (n = 73). Figure S1. Scatter plots showing the partial correlation between serum thyroid hormones and Aβ deposition, and tau deposition. Figure S2. Sensitivity analysis: Moderating effects of thyroid hormones on the relationships between Aβ and tau deposition, excluding an outlier (n = 73). [file 13195_2024_1534_MOESM1_ESM.docx]

**Supplementary materials**

**Table S1.** Demographic and clinical characteristics of participants according to fT4 subgroups

| Characteristics | Low fT4 subgroup  (n=37) | High fT4 subgroup  (n=37) | t, χ² | p-value |
| --- | --- | --- | --- | --- |
| Age (years) | 69.38 (7.52) | 69.86 (7.51) | -0.278 | 0.781 |
| Education (years) | 11.54 (4.28) | 11.46 (4.46) | 0.080 | 0.937 |
| Gender, female (%) | 26 (70.3) | 21 (56.8) | 1.458 | 0.227 |
| CDR | 0 | 0 |  |  |
| APOE ε4 allele(+) (%) | 5 (13.5) | 9 (24.3) | 1.410 | 0.235 |
| GDS | 5.27 (5.38) | 5.08 (4.57) | 0.163 | 0.871 |
| VRS | 1.19 (1.13) | 1.03 (0.83) | 0.704 | 0.484 |
| Neuropsychological tests |  |  |  |  |
| MMSE-KC score | 27.24 (2.29) | 27.08 (1.95) | 0.328 | 0.744 |
| CERAD-K total score | 72.86 (10.32) | 72.35 (9.54) | 0.222 | 0.825 |
| Global Aβ deposition (SUVR) | 1.29 (0.31) | 1.33 (0.39) | -0.428 | 0.670 |
| Amyloid positivity (%) | 11 (29.7) | 12 (32.4) | 0.063 | 0.802 |
| IT tau deposition (SUVR) | 1.45 (0.24) | 1.41 (0.30) | 0.568 | 0.572 |
| Tau positivity (%) | 10 (27.0) | 8 (21.6) | 0.294 | 0.588 |
| T3 (mg/dl) | 102.08 (18.69) | 104.54 (16.61) | -0.598 | 0.552 |
| TSH (μIU/ml) | 2.46 (1.70) | 1.99 (1.34) | 1.326 | 0.189 |
| fT4 (ng/dl) | 1.02 (0.08) | 1.26 (0.11) | -10.505 | 0.000 |
| fT3 (pg/ml) | 3.00 (0.29) | 3.14 (0.38) | -1.865 | 0.066 |

**Notes:** Data for continuous variables are presented as a mean (SD). Categorical variables are presented as N (%). Continuous variables were tested for differences between the two groups using independent samples t-tests, while categorical variables were tested using chi-square tests.

**Abbreviations:** CDR Clinical Dementia Rating, APOE Apolipoprotein E, GDS Geriatric Depression Scale, VRS Vascular Risk Score, MMSE-KC Mini-Mental State Examination in the Korean version of CERAD Assessment Packet, CERAD-K Korean version of the Consortium to Establish a Registry for Alzheimer’s Disease Assessment Packet, Aβ beta-amyloid, SUVR standardized uptake value ratio, IT inferior temporal, T3 triiodothyronine, T4 thyroxine, TSH thyroid-stimulating hormone

**Table S2.** Demographic and clinical characteristics of participants according to fT3 subgroups

| Characteristics | Low fT3 subgroup  (n=37) | High fT3 subgroup  (n=37) | t, χ² | p-value |
| --- | --- | --- | --- | --- |
| Age (years) | 69.35 (8.00) | 69.89 (7.00) | -0.309 | 0.758 |
| Education (years) | 11.59 (4.62) | 11.41 (4.11) | 0.186 | 0.853 |
| Gender, female (%) | 27 (73.0) | 20 (54.1) | 2.857 | 0.091 |
| CDR | 0 | 0 |  |  |
| APOE ε4 allele(+) (%) | 5 (13.5) | 9 (24.3) | 1.410 | 0.235 |
| GDS | 5.76 (5.04) | 4.59 (4.88) | 1.008 | 0.317 |
| VRS | 1.27 (1.10) | 0.95 (0.85) | 1.423 | 0.159 |
| Neuropsychological tests |  |  |  |  |
| MMSE-KC score | 27.24 (1.92) | 27.08 (2.31) | 0.328 | 0.744 |
| CERAD-K total score | 71.78 (10.44) | 73.43 (9.34) | -0.716 | 0.476 |
| Global Aβ deposition (SUVR) | 1.29 (0.31) | 1.32 (0.39) | -0.355 | 0.724 |
| Amyloid positivity (%) | 11 (29.7) | 12 (32.4) | 0.063 | 0.802 |
| IT tau deposition (SUVR) | 1.45 (0.22) | 1.40 (0.31) | 0.757 | 0.451 |
| Tau positivity (%) | 11 (29.7) | 7 (18.9) | 1.175 | 0.278 |
| T3 (mg/dl) | 95.43 (13.68) | 111.19 (17.71) | -4.283 | 0.000 |
| TSH (μIU/ml) | 2.38 (1.57) | 2.07 (1.50) | 0.843 | 0.402 |
| fT4 (ng/dl) | 1.10 (0.13) | 1.18 (0.17) | -2.433 | 0.017 |
| fT3 (pg/ml) | 2.81 (0.21) | 3.33 (0.24) | -9.822 | 0.000 |

**Notes:** Data for continuous variables are presented as a mean (SD). Categorical variables are presented as N (%). Continuous variables were tested for differences between the two groups using independent samples t-tests, while categorical variables were tested using chi-square tests.

**Abbreviations:** CDR Clinical Dementia Rating, APOE Apolipoprotein E, GDS Geriatric Depression Scale, VRS Vascular Risk Score, MMSE-KC Mini-Mental State Examination in the Korean version of CERAD Assessment Packet, CERAD-K Korean version of the Consortium to Establish a Registry for Alzheimer’s Disease Assessment Packet, Aβ beta-amyloid, SUVR standardized uptake value ratio, IT inferior temporal, T3 triiodothyronine, T4 thyroxine, TSH thyroid-stimulating hormone

**Table S3.** Sensitivity analysis: Partial correlation between serum thyroid hormones and Aβ deposition, and tau deposition in euthyroid participants

|  |  | T3 | TSH | fT4 | fT3 |
| --- | --- | --- | --- | --- | --- |
| Aβ deposition | correlation | 0.058 | -0.004 | -0.002 | -0.016 |
| (n=260) | p-value | 0.354 | 0.950 | 0.974 | 0.793 |
| Tau deposition | correlation | 0.073 | -0.019 | 0.133 | -0.032 |
| (n=65) | p-value | 0.575 | 0.883 | 0.304 | 0.805 |

**Notes:** Partial correlation analysis used age, sex, and APOE ε4 positivity as the covariates.

**Abbreviations:** Aβ beta-amyloid, T3 triiodothyronine, T4 thyroxine, TSH thyroid-stimulating hormone

**Table S4.** Sensitivity analysis: Interaction effects of serum thyroid hormones with global Aβ deposition on inferior temporal tau deposition in euthyroid participants (n=65)

|  | B (95% CI) | SE | p-value |
| --- | --- | --- | --- |
| Model 1^a^ |  |  |  |
| Global Aβ deposition | 0.452 (0.285-0.618) | 0.083 | 0.000 |
| Model 2^b^ |  |  |  |
| Global Aβ deposition x T3 | 0.002 (-0.010-0.014) | 0.006 | 0.724 |
| Global Aβ deposition x TSH | -0.065 (-0.230-0.100) | 0.083 | 0.435 |
| Global Aβ deposition x fT4 | 1.395 (0.730-2.060) | 0.332 | 0.000 |
| Global Aβ deposition x fT3 | 0.763 (0.341-1.185) | 0.211 | 0.001 |

**Notes:** ^a^ A multiple linear regression model for IT tau deposition included Global Aβ deposition as an independent variable, after adjusting for age, sex, and APOE ε4 positivity.

^b^ Multiple linear regression models for IT tau deposition included serum thyroid hormones and Global Aβ as interactive predictors, after adjusting for age, sex, and APOE ε4 positivity.

Summary of the model: IT tau ~ Global Aβ deposition x Thyroid hormone + Thyroid hormone + Global Aβ deposition + age + sex + APOE ε4 positivity

**Abbreviations:** Aβ beta-amyloid, T3 triiodothyronine, T4 thyroxine, TSH thyroid-stimulating hormone, IT inferior temporal, APOE Apolipoprotein E, CI confidence interval, SE standard error

**Table S5.** Sensitivity analysis: Partial correlation between serum thyroid hormones and Aβ deposition, and tau deposition after including VRS as an additional covariate

|  |  | T3 | TSH | fT4 | fT3 |
| --- | --- | --- | --- | --- | --- |
| Aβ deposition | correlation | 0.070 | -0.008 | -0.050 | -0.012 |
| (n=291) | p-value | 0.236 | 0.889 | 0.403 | 0.838 |
| Tau deposition | correlation | 0.086 | -0.211 | -0.012 | -0.075 |
| (n=74) | p-value | 0.477 | 0.079 | 0.921 | 0.538 |

**Notes:** Partial correlation analysis used age, sex, APOE ε4 positivity, and VRS as the covariates.

**Abbreviations:** Aβ beta-amyloid, VRS Vascular Risk Score, T3 triiodothyronine, T4 thyroxine, TSH thyroid-stimulating hormone

**Table S6.** Sensitivity analysis: Interaction effects of serum thyroid hormones with global Aβ deposition on inferior temporal tau deposition after including VRS as an additional covariate (n=74)

|  | B (95% CI) | SE | p-value |
| --- | --- | --- | --- |
| Model 1^a^ |  |  |  |
| Global Aβ deposition | 0.491 (0.325-0.657) | 0.083 | 0.000 |
| Model 2^b^ |  |  |  |
| Global Aβ deposition x T3 | 0.002 (-0.007-0.012) | 0.005 | 0.643 |
| Global Aβ deposition x TSH | -0.069 (-0.150-0.011) | 0.040 | 0.091 |
| Global Aβ deposition x fT4 | 1.025 (0.377-1.672) | 0.324 | 0.002 |
| Global Aβ deposition x fT3 | 0.729 (0.321-1.137) | 0.204 | 0.001 |

**Notes:** ^a^ A multiple linear regression model for IT tau deposition included Global Aβ deposition as an independent variable, after adjusting for age, sex, APOE ε4 positivity, and VRS.

^b^ Multiple linear regression models for IT tau deposition included serum thyroid hormones and Global Aβ as interactive predictors, after adjusting for age, sex, APOE ε4 positivity, and VRS.

Summary of the model: IT tau ~ Global Aβ deposition x Thyroid hormone + Thyroid hormone + Global Aβ deposition + age + sex + APOE ε4 positivity + VRS

**Abbreviations:** Aβ beta-amyloid, VRS Vascular Risk Score, T3 triiodothyronine, T4 thyroxine, TSH thyroid-stimulating hormone, IT inferior temporal, APOE Apolipoprotein E, CI confidence interval, SE standard error

**Table S7.** Sensitivity analysis: Interaction effects of thyroid hormones with Aβ deposition on tau deposition, excluding an outlier (n=73)

|  | B (95% CI) | SE | p-value |
| --- | --- | --- | --- |
| Model 1^a^ |  |  |  |
| Global Aβ deposition | 0.354 (0.187-0.522) | 0.084 | 0.000 |
| Model 2^b^ |  |  |  |
| Global Aβ deposition x T3 | 0.005 (-0.004-0.014) | 0.005 | 0.249 |
| Global Aβ deposition x TSH | -0.045 (-0.124-0.034) | 0.040 | 0.259 |
| Global Aβ deposition x fT4 | 0.125 (-0.984-1.234) | 0.555 | 0.823 |
| Global Aβ deposition x fT3 | 0.557 (0.150-0.963) | 0.204 | 0.008 |

**Notes:** ^a^ A multiple linear regression model for IT tau deposition included Global Aβ deposition as an independent variable, after adjusting for age, sex, and APOE ε4 positivity.

^b^ Multiple linear regression models for IT tau deposition included serum thyroid hormones and global Aβ as interactive predictors, after adjusting for age, sex, and APOE ε4 positivity.

Summary of the model: IT tau ~ Global Aβ deposition x Thyroid hormone + Thyroid hormone + Global Aβ deposition + age + sex + APOE ε4 positivity

**Abbreviations:** Aβ beta-amyloid, T3 triiodothyronine, T4 thyroxine, TSH thyroid-stimulating hormone, IT inferior temporal, APOE Apolipoprotein E, CI confidence interval, SE standard error

**Table S8.** Sensitivity analysis: Relationship between Aβ and tau deposition in subgroups based on thyroid hormone levels, excluding an outlier (n=73)

|  | B (95% CI) | SE | R^2^ | p-value |
| --- | --- | --- | --- | --- |
| Low fT4 (fT4<1.12) | 0.326 (-0.001-0.653) | 0.161 | 0.182 | 0.051 |
| High fT4 (fT4≥1.12) | 0.375 (0.183-0.568) | 0.094 | 0.434 | 0.000 |
| Low fT3 (fT3<3.05) | 0.173 (-0.076-0.421) | 0.122 | 0.215 | 0.167 |
| High fT3 (fT3≥3.05) | 0.518 (0.303-0.733) | 0.105 | 0.491 | 0.000 |

**Notes:** Multiple linear regression models for IT tau deposition included global Aβ deposition as an independent variable, after adjusting for age, sex, and APOE ε4 positivity.

**Abbreviations:** Aβ beta-amyloid, T3 triiodothyronine, T4 thyroxine, IT inferior temporal, APOE Apolipoprotein E, CI confidence interval, SE standard error

**Figure S1.** Scatter plots showing the partial correlation between serum thyroid hormones and Aβ deposition, and tau deposition

**
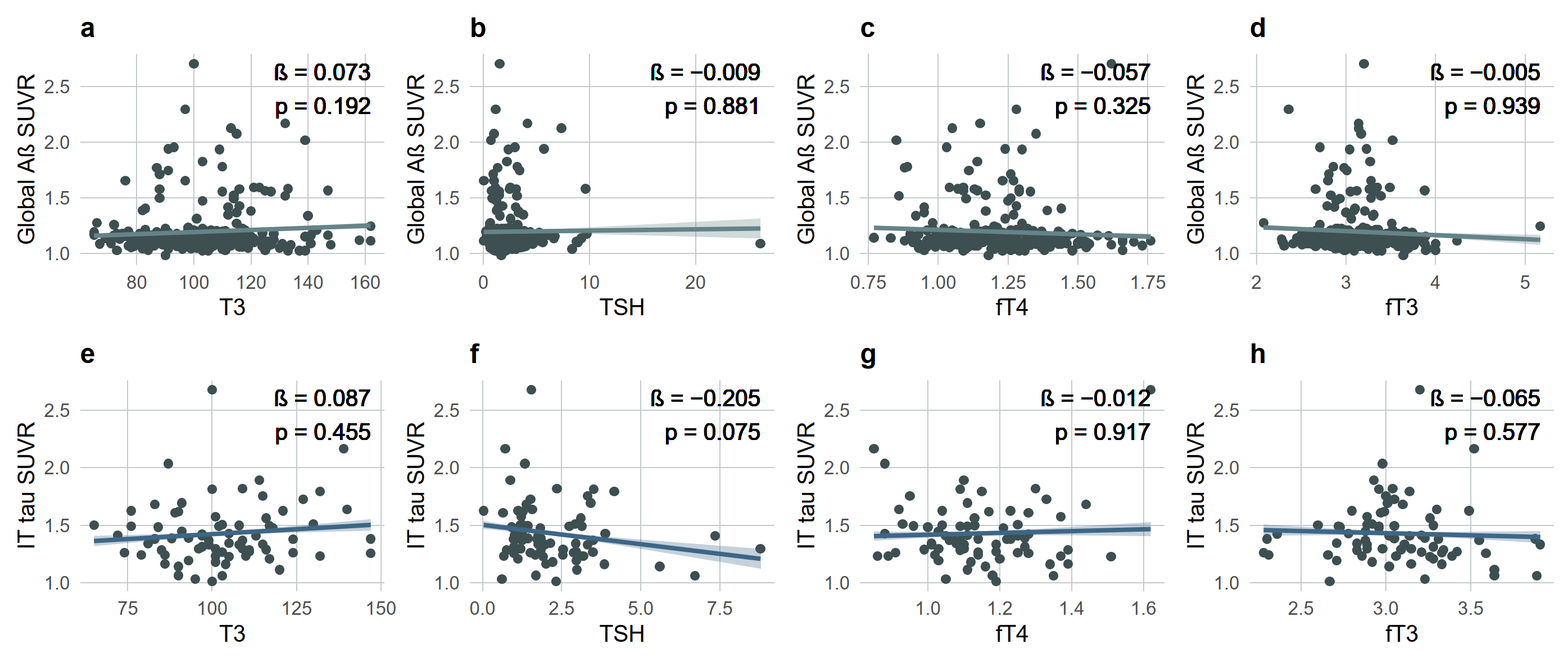
**

**Notes:** Partial correlation analysis used age, sex, and APOE ε4 positivity as the covariates.

**Abbreviations:** Aβ beta-amyloid, IT inferior temporal, SUVR standardized uptake value ratio, T3 triiodothyronine, T4 thyroxine, TSH thyroid-stimulating hormone, APOE Apolipoprotein E

**Figure S2.** Sensitivity analysis: Moderating effects of thyroid hormones on the relationships between Aβ and tau deposition, excluding an outlier (n=73)

**
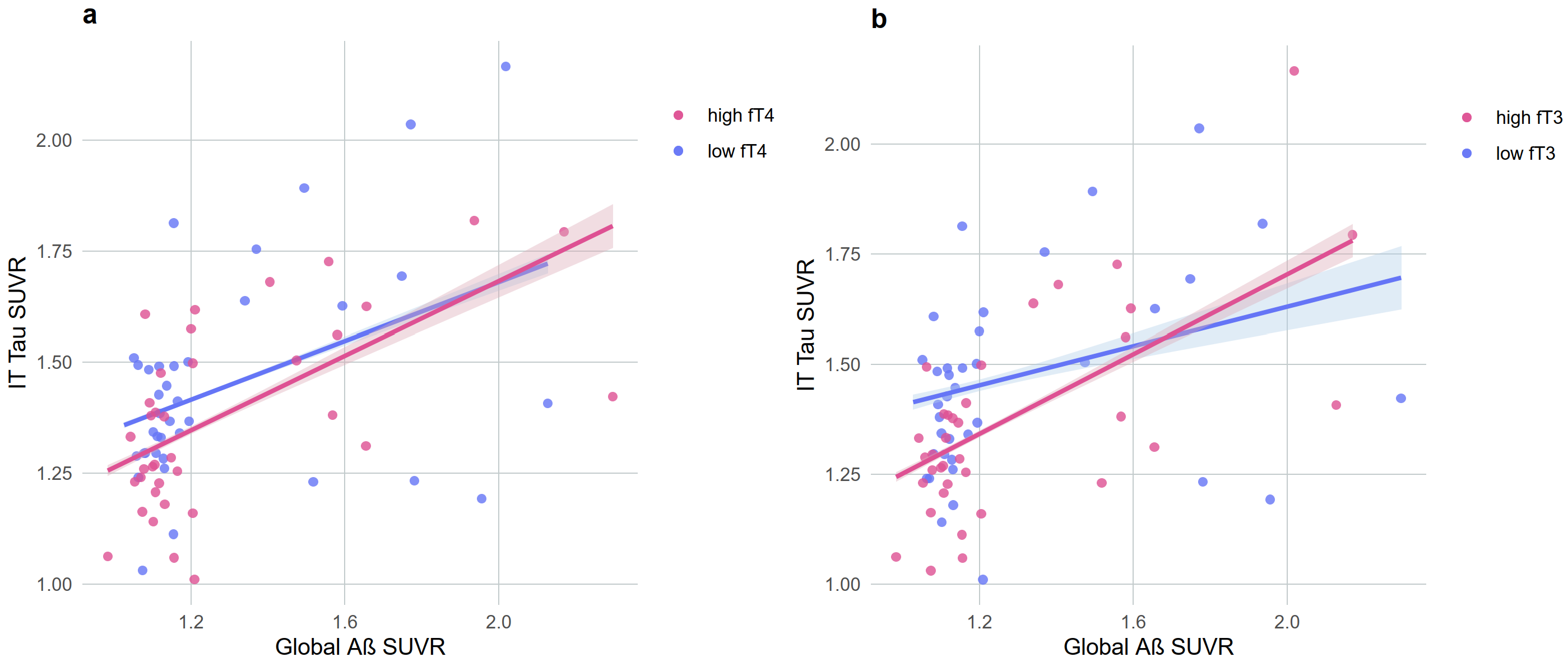
**

**Notes:** To effectively demonstrate the moderating effects, participants were divided into two subgroups based on thyroid hormone levels. Each line represents a regression line for IT tau deposition, with global Aβ deposition as the independent variable, controlling for age, sex, and APOE ε4 positivity. The shaded regions demonstrate the 95% confidence intervals of the regression lines.

**Abbreviations:** Aβ beta-amyloid, IT inferior temporal, SUVR standardized uptake value ratio, T3 triiodothyronine, T4 thyroxine, APOE Apolipoprotein E
